# A company experience of the first MoCA pilot project

**DOI:** 10.1186/1750-1172-9-S1-O26

**Published:** 2014-11-11

**Authors:** Wills Hughes-Wilson

**Affiliations:** 1 Sobi, SE-112 76 Stockholm, Sweden

## Background

Pricing and reimbursement authorities often lack sufficient information to decide about Orphan Medicinal Products (OMPs), because of pricing, high levels of uncertainty and small datasets, amongst others. EU Member States share similar challenges as they seek to include OMPs appropriately in their healthcare systems.

The “Process on Corporate Responsibility in the field of Pharmaceuticals”, launched in 2010 under the Belgium EU Presidency, included a project on a “Mechanism of Coordinated Access to Orphan Drugs” (MoCA), to explore whether a collaborative approach could create opportunities for more timely, sustainable and equitable access to OMPs. Twelve EU Member States, industry, patients’ representatives and other stakeholders developed a potential mechanism for such cooperation in the MoCA Recommendations. EU Member States adopted these as part of the formal conclusions of the Process on Corporate Responsibility on 17 April 2013. The Recommendations identified points where voluntary cooperation could smooth the process of evaluation, by sharing information and data along a coordinated, dialogue-based pathway; as well as a first draft “Transparent Value Framework” (TVF) to provide a possible structure for national pricing and reimbursement discussions.

**Figure 1:**
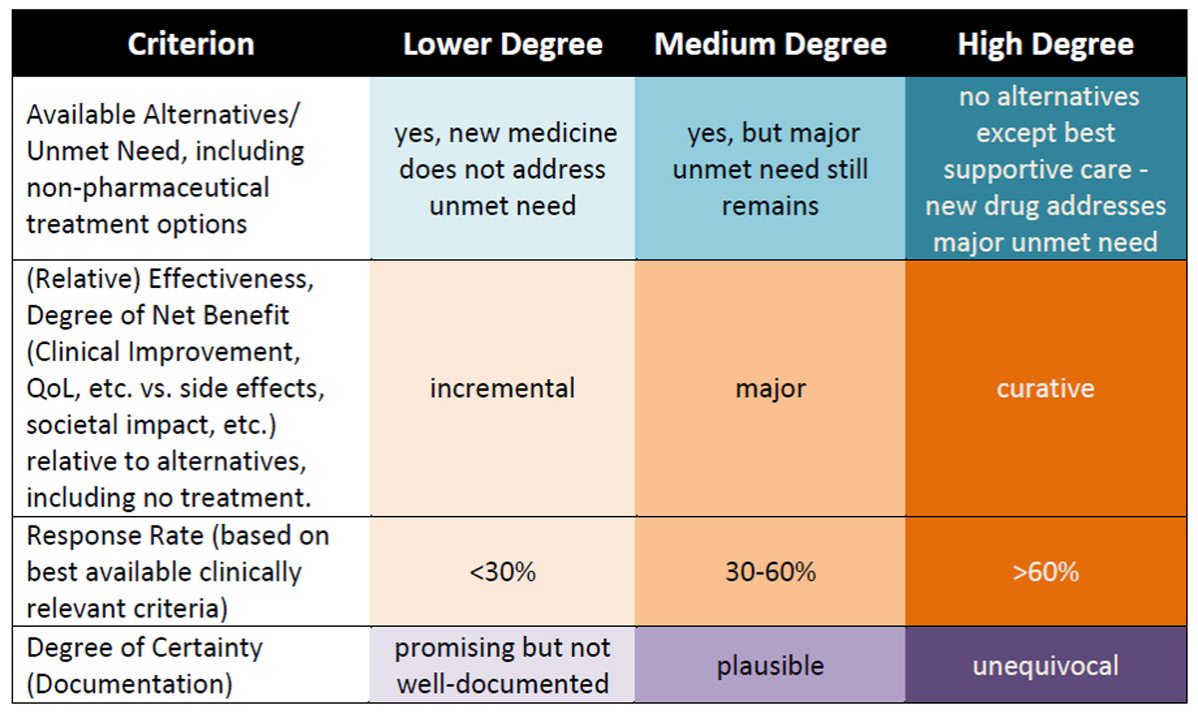
An example of a proposed Transparent Value Framework

## From theory to practice: the MoCA pilot projects

The Medicines Evaluation Committee of the European Social Insurance Platform (MEDEV) initiated pilot projects in July 2013. Companies were invited to participate with OMPs at any stage of development, with the objective of testing the different elements in the Recommendations. Sobi signalled its interest to participate.

**Figure 2:**
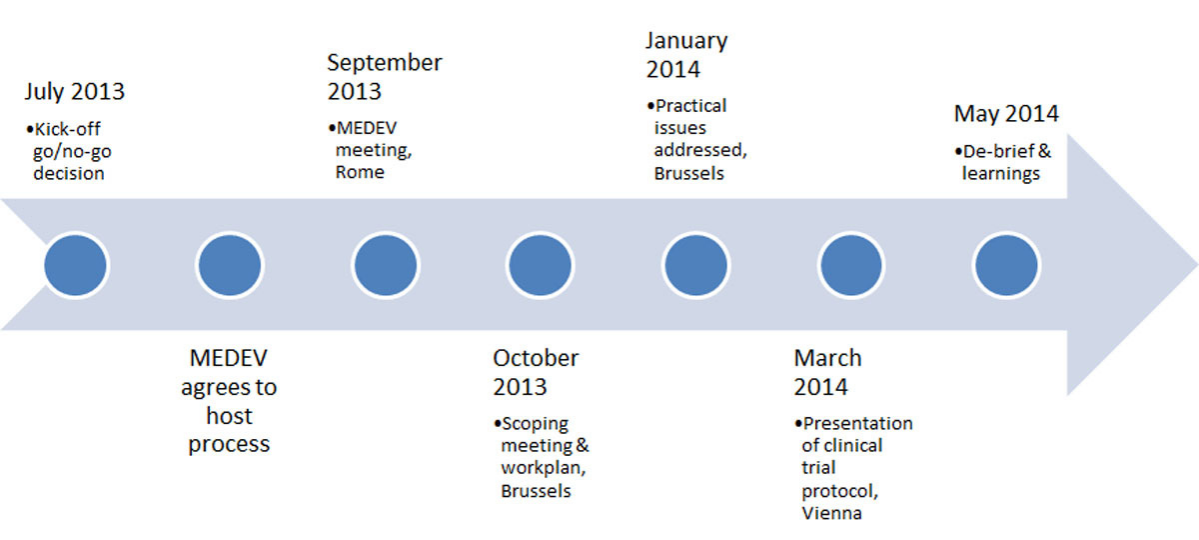
Timeline of MoCA pilot project July 2013-May 2014

Seven EU Member States volunteered to join the collaboration, together with European Organisation for Rare Diseases (EURORDIS) and two patient groups representing the therapeutic area. The participants agreed potential areas for collaboration, created a timeline of meeting and topics for shared discussion (Figure 2), also including other stakeholders.

## Sobi’s experience of participating in the MoCA pilot project

The May 2014 evaluation provided opportunities to review the MoCA dialogue’s ability to deliver on its objectives and where more work needs to be done. Sobi experienced the MoCA as an important and highly relevant forum, providing the opportunity for managed, prospective, multi-stakeholder, trust-based dialogue, which progressively reduces uncertainty and allows sponsors to “design-in” payer-driven elements into development programmes and availability. It is based on voluntary participation of all participants so is highly flexible. It gives national payer bodies information to help them in their national providing and reimbursement decisions.

## Conclusions

The first pilot was a success in testing the practical elements of a collaborative dialogue. Further projects will further test the Recommendations and the MoCA’s ability to contribute to access to OMPs timely, equitably and sustainably. The participants in the MoCA pilot are planning to write up their experiences with an aim to publish their joint findings to date.

